# Towards the identification of protein complexes and functional modules by integrating PPI network and gene expression data

**DOI:** 10.1186/1471-2105-13-109

**Published:** 2012-05-23

**Authors:** Min Li, Xuehong Wu, Jianxin Wang, Yi Pan

**Affiliations:** 1 School of Information Science and Engineering, Central South University, China; 2 State Key Laboratory of Medical Genetics, Central South University, China; 3 Department of Computer Science, Georgia State University, USA

## Abstract

**Background:**

Identification of protein complexes and functional modules from protein-protein interaction (PPI) networks is crucial to understanding the principles of cellular organization and predicting protein functions. In the past few years, many computational methods have been proposed. However, most of them considered the PPI networks as static graphs and overlooked the dynamics inherent within these networks. Moreover, few of them can distinguish between protein complexes and functional modules.

**Results:**

In this paper, a new framework is proposed to distinguish between protein complexes and functional modules by integrating gene expression data into protein-protein interaction (PPI) data. A series of time-sequenced subnetworks (TSNs) is constructed according to the time that the interactions were activated. The algorithm TSN-PCD was then developed to identify protein complexes from these TSNs. As protein complexes are significantly related to functional modules, a new algorithm DFM-CIN is proposed to discover functional modules based on the identified complexes. The experimental results show that the combination of temporal gene expression data with PPI data contributes to identifying protein complexes more precisely. A quantitative comparison based on f-measure reveals that our algorithm TSN-PCD outperforms the other previous protein complex discovery algorithms. Furthermore, we evaluate the identified functional modules by using “Biological Process” annotated in GO (Gene Ontology). The validation shows that the identified functional modules are statistically significant in terms of “Biological Process”. More importantly, the relationship between protein complexes and functional modules are studied.

**Conclusions:**

The proposed framework based on the integration of PPI data and gene expression data makes it possible to identify protein complexes and functional modules more effectively. Moveover, the proposed new framework and algorithms can distinguish between protein complexes and functional modules. Our findings suggest that functional modules are closely related to protein complexes and a functional module may consist of one or multiple protein complexes. The program is available at http://netlab.csu.edu.cn/bioinfomatics/limin/DFM-CIN/index.html.

## Background

Recent advances in biotechnology have resulted in a large amounts of protein-protein interaction (PPI) data. Modeling and clustering PPI networks with simple graphs makes it possible for us to understand the basic components and organization of cell machinery from the network level[1]. One of the most important challenges in the post-genomic era is to analyze the complex networks of PPIs and detect protein complexes or functional modules from them. Over the past decade, many computational methods have been proposed for clustering PPI networks, such as G-N [2], MCODE[3], RNSC[4], LCMA[5], DPClus [6], MoNet [7], IPCA [8], COACH [9], and SPICi [10].

While significant progress has been made in computational methods, there are two major challenges in clustering PPI networks. One of the challenges is that the conventional clustering methods generally considered the PPI network as a static graph and overlooked the dynamics inherent within these networks. This is mainly because that the widely used large-scale technologies for determining PPIs, such as yeast two-hybrid and TAP-MS, do not provide spatial, temporal or contextual information for the predicted PPIs [11]. In fact, a PPI network is not a static but a dynamic entity, so whether or not a protein is expressed is intrinsically controlled by different regulatory mechanisms through time and space [12,13]. Recently, studies on network dynamics have begun to attract researchers’ attentions[11,14]. Of course, biologists have studied dynamics in biological systems for many years. However, their efforts generally focused on individual genes or proteins as well as specific interactions in limited contexts. With the accumulation of PPI and transcriptome data, the integration of gene expression profiles with PPIs provides new way of uncovering the dynamics of PPI networks [15,16].

Jansen et al. [17] first investigated the relationship of PPI interactions with mRNA expression levels and scored expression activity in complexes. Tornow and Mewes[18] used the superparamagnetic approach to evaluate the multi-data correlations and constructed a graph of co-expressed genes for detecting functional modules. Han et al. [12] analyzed the PPI network of yeast, and they uncovered two types of hub proteins: “party” hubs and “date” hubs. Recently, Taylor et al.[19] also proposed another two types of hub proteins: intermodular hubs and intramodular hubs, and they investigated the modularity of human PPI networks in two breast cancer patient groups. Xue et al. [20] analyzed the dynamic modular structure of the human PPI network in their aging study. Lu et al. [21] proposed a simple hierarchical clustering algorithm for analyzing the dynamic organization of biological networks by integrating the yeast PPI interaction data, the global subcellular localization data and the integrated expression profile data. Cline et al. [22] described how to integrate biological networks and gene expression data by using Cytoscape. Maraziotis et al. [23] presented a method to detect dense subnetworks in a weighted graph that was constructed by using the gene expression information. Cho et al. [24] also introduced an algorithm based on informative protein selection from a weighted graph where the weight was calculated by using co-expressional profiles. More recently, Luo et al. [25] explored special kinds of protein complexes by integrating transcription regulation data, gene expression data and PPI data at the systems biology level. Hegde et al. [26] proposed an approach for studying an organism at the systems level by integrating genome-wide functional linkages and the gene expression data. De Lichtenberg U et al.[27] combined the subcellular localization data, gene expression data and PPI network to extract a temporal protein interaction network of the yeast mitotic cell cycle. Komurov and White[28] used gene expression data to classify dynamic proteins which are expressed periodically and static proteins which are expressed all the time, and furthermore identified dynamic modules and static modules on a static PPI network. Similar techniques were also applied to the identification of disease-related genes or modules [19,29]. All these works have made significant progress in the integration of co-expression information and PPI networks. However, only a few of them focused on the identification of protein complexes or functional modules. Some of them only used gene expression information to construct weighted PPI network which was still static.

Another challenge in clustering PPI networks is how to distinguish between protein complexes and functional modules. Up to now, little progress has been made on this point. Most clustering methods based on PPI networks detected both protein complexes and functional modules without distinguishing between them because they disregard interaction dynamics. How closely are functional modules related to protein complexes? What are the differences between them? Spirin and Mirny have argued their differences from the concepts that protein complexes are groups of proteins interacting with each other at the same time, and functional modules, by contrast, are groups of proteins participating in a particular cellular process while binding to each other at a different times[30]. Though Spirin and Mirny believed that it was very important to distinguish between protein complexes and functional modules, they did not distinguish between the two because that they lacked temporal and spatial information on the analyzed PPIs. Recently, Lu et al[21] proposed to make this distinction by integrating PPI data with the added subcellular localization and expression profile data. They investigated the relationship between protein complexes and functional modules and revealed that a functional module generally consists of proteins that participate in a common biological process, and that protein complexes form the intersections of co-localized and co-expressed protein groups that are usually included in the functional modules[21].

In this paper, we will not go as far as what the conventional clustering algorithms have focused on but rather try to propose a framework to detect and distinguish between protein complexes and functional modules. In other words, we will not only explore protein complexes and functional modules but also study their relationships. Considering the fact that proteins in a complex interact with each other at the same time, we constructed a serial of time-sequenced subnetworks by integrating gene expression data into PPI data. These time-sequenced subnetworks show dynamic changes in the original network. Thus, we call these time-sequenced subnetworks together as a dynamic PPI network. An improved algorithm TSN-PCD, developed from our previous algorithm HC-PIN[31], is proposed to identify protein complexes from the dynamic PPI network. Applying TSN-PCD to a dynamic PPI network of *S.cerevisiae*, we found that many proteins were found in a multitude of complexes rather than a single complex. Here, we would like to ask whether two protein complexes interact with each other through their common proteins. Moreover, what is the underlying machine between protein complexes and functional modules. To answer these questions, we constructed a complex-complex interaction network and proposed an algorithm, DFM-CIN, for detecting functional modules from it.

In the case of identifying protein complexes, we found more known protein complexes are recalled after the combination of temporal gene expression data with PPI data. We also found not only the combination of temporal gene expression data with PPI data but also the algorithm TSN-PCD contribute to detecting protein complexes more precisely. A quantitative comparison based on *f *-measure reveals that our algorithm TSN-PCD outperforms six other previously proposed protein complex discovery algorithms: MCL[32,33]), MCODE[3], CPM[34], COACH[9], SPICI[10], and HC-PIN[31]. Furthermore, we evaluated the identified functional modules by using “Biological Process” annotated in GO (Gene Ontology) and found most of them participated in a special biological process. Additionally, we even found the relationship between protein complexes and functional modules. Our findings suggest that functional modules are closely related to protein complexes and a functional module may consist of one or multiple protein complexes.

## Methods

### A framework for detecting protein complex and functional module

When clustering PPI networks, people seldom distinguish between protein complex and function modules. However, they are not the same thing. The main difference between them is that protein complexes occur at the same time, functional modules, generally function at different times. Spirin and Mirny [30] have discussed the differences between protein complex and functional module from biological view. According to Spirin and Mirny’s perspective, we defined protein complex and function module as follows: (1)Protein complexes are groups of proteins that interact with each other at the same time and place, forming single multi-molecular machine, such as AP-2 adaptor complex, DNA polymerase epsilon complex, Dig1p/St12p/Dig2p complex, SAS complex. (2)Functional modules, in contrast, consist of proteins that participate in a particular cellular process while binding each other at a different time and place, such as the CDK/cyclin module responsible for cell-cycle progression, the yeast pheromone response pathway, MAP signaling cascades. In this paper, we can not only predict protein complexes and functional modules but also distinguish them.

**Figure 1:**
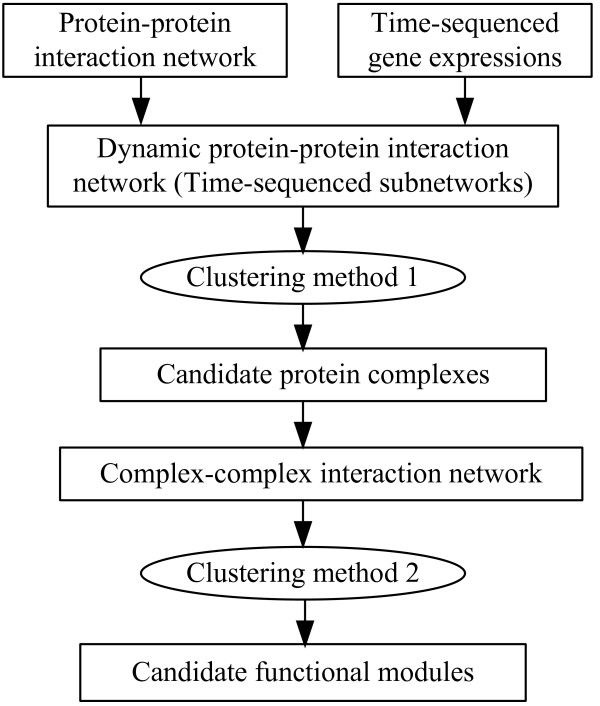
**A framework for detecting protein complex and functional modules based on the combination of a PPI network and time-sequenced gene expressions.** Clustering method 1 is used for predicting protein complexes while clustering method 2 is used for mining functional modules according to the identified complexes. In this framework, each previous clustering algorithm can be used as a candidate of clustering method 1 for identifying protein complexes as well as clustering method 2 for identifying functional modules.

Previous studies [11,15,16,21,26] have shown that by integrating co-expression information into PPI networks, one can acquire the dynamic features among networks. The first question, perhaps, is how to construct a dynamic PPI network by using these data. Here, we construct a dynamic PPI network by splitting the original static network into a serial of time-sequenced subnetworks (TSNs) as we have done in [16]. When generating TSNs, a fixed threshold value is used to filter gene products at each time point. Only the transcripts whose expression levels are greater than a fixed threshold value are remained. By combined the filtered transcripts and PPI network data, the TSNs are created. In each subnetwork TSN, all the interactions are activated at the same time. Then, a clustering method can be applied on each subnetwork TSN to explore protein complexes. In this case, proteins in every identified protein complex will interact with each other at the same time. Here, we will highlight a new framework for detecting and distinguishing between protein complexes and functional modules, as shown in Figure 1. In Figure 1, each previous clustering algorithm can be used as a candidate of clustering method 1 for identifying protein complexes.

Preliminary observation of protein complexes and functional modules has indicated that while protein complexes occur at the same time, functional modules, generally function at different times. The former are usually included in the latter [21,30]). According to the close relationship between protein complexes and functional modules, as well as the obvious difference between them, we propose to discover functional modules based on the identified protein complexes. A complex-complex interaction network is constructed based on analyzing the relationship among the identified protein complexes. In the complex-complex interaction network, each vertex represents a protein complex and each edge represents the relationship of two protein complexes. Then, a clustering algorithm can be applied on the complex-complex interaction network to explore functional modules. Different clustering algorithms can also be used here. To distinguish it from the protein complex discovery algorithm, we mark it as clustering method 2 in Figure 1. Next, we will discuss two specific algorithms for identifying protein complexes and functional modules, respectively.

### TSN-PCD: Time-sequenced network-based protein complex discovery algorithm

**Figure 2:**
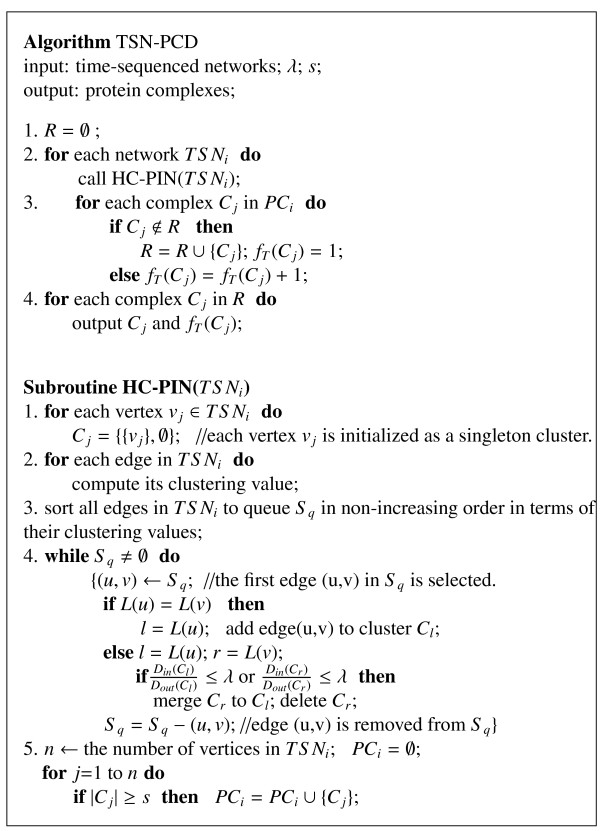
**The description of algorithm TSN-PCD.** One of the inputs for TSN-PCD is the subnetworks which are generated from the combination of the PPI network and gene expression data. HC-PIN is used here to predict protein complexes from the subnetworks with two parameter *λ*and s. The output of TSN-PCD is the predicted protein complexes.

Based on the combination of PPI network and time-sequenced gene expressions, a serial of time-sequenced subnetworks (TSNs) is constructed. The TSNs-based protein complex discovery algorithm is named as TSN-PCD. The description of algorithm TSN-PCD is shown in Figure 2.

Given *k* gene expression data at *k* different times, *k* time-sequenced subnetworks (TSNs) are generated. These *k* time-sequenced subnetworks form a dynamic process of the PPI network. Each TSN is a subnetwork of the original PPI network. All interactions among proteins in each TSN happen at the same time. Then, a clustering can be performed on each TSN to produce protein complexes. Here, the same strategies used in HC-PIN[31] are adopted to generate protein complexes in each TSN. For a TSN_*i*_, the vertices in it are initialized as singleton clusters at first. Then, the clustering value of each edge in it is calculated. The clustering value of an edge (*uv*) is defined as:

(1) $$\mathrm{ECV}(u,v)=\frac{\underset{k\in I_{u,v}}{\sum }w(u,k)\cdot \underset{k\in I_{u,v}}{\sum }w(v,k)}{\underset{s\in N_{u}}{\sum }w(u,s)\cdot \underset{t\in N_{v}}{\sum }w(v,t)}$$

where *N*_*u*_ denotes the set of neighbors of vertex *uN*_*v*_ denotes the set of neighbors of vertex *v*, and _*Iu*,*v*_ denotes the set of common vertices in *N*_*u*_ and *N*_*v*_ (i.e. $I_{u,v}=N_{u}\cap N_{v}$).

All the edges in TSN_*i*_ are queued into _*Sq*_in a non-increasing order in terms of their clustering values. Then, different clusters are constantly reassembled into larger clusters by gradually removing edges from the queue. The basic idea is that the higher clustering value the edge has, the more likely its two vertices are inside the same protein complex. Finally, the clusters which consist of no less than *s* proteins are produced as protein complexes.

In this step, the previous clustering algorithms used in static PPI networks can also be used here. As our proposed algorithm HC-PIN outweighs other clustering algorithms in most cases. We thus use it to predict protein complexes with the recommended parameter *λ*=1.0.

**Figure 3:**
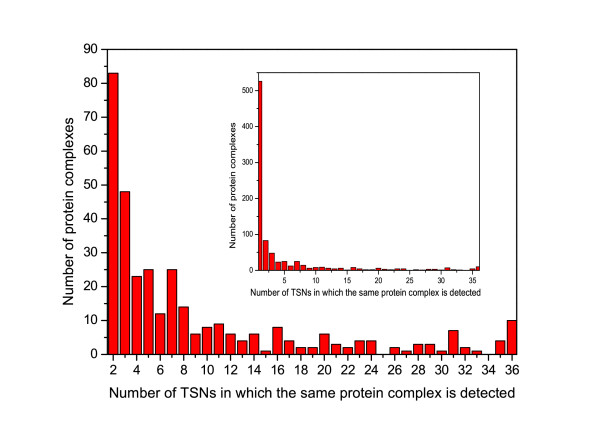
**The distribution of frequencies of protein complexes detected in TSNs.** There are 865 different protein complexes detected by TSN-PCD, in which ∼60*%*protein complexes are explored only in one TSN and ∼24*%*protein complexes are discovered in more than three TSNs as shown in a sub-figure with one TSN in the top right corner of Figure 3.

A protein complex may exist in only one TSN or in multiple TSNs. Let *T*={_*t*1_,_*t*2_,⋯,_*tk*_} be a time period. Then, the frequency _*fT*_(_*Cj*_) of a protein complex _*Cj*_ is defined as the number of TSNs in which protein complex _*Cj*_exists. Figure 3 shows the frequencies of protein complexes detected in these TSNs. There are 865 different protein complexes detected, in which ∼60*%* protein complexes are explored only in one TSN and ∼24*%*protein complexes are discovered in more than three TSNs. The frequency of each identified protein complex and the information of subnetworks in which the protein complex is included are available from Additional file 1.

### DFM-CIN: detecting functional modules from the complex-complex interaction network

**Figure 4:**
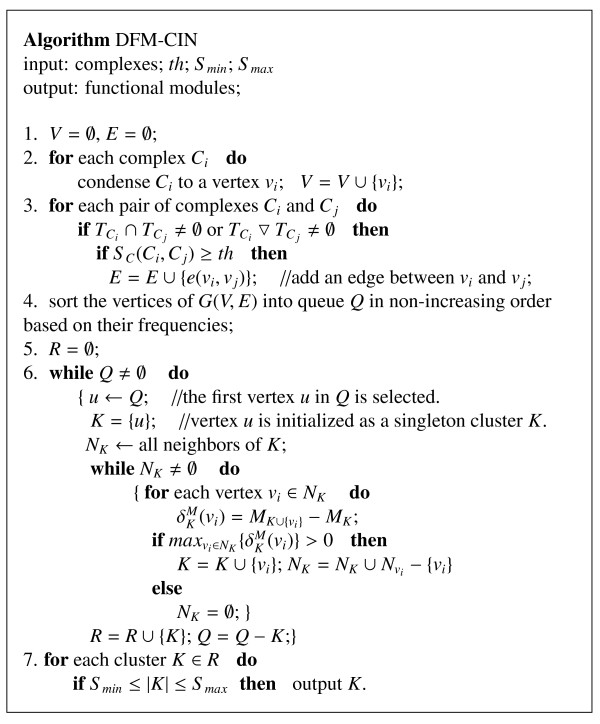
**The description of algorithm DFM-CIN.** The inputs of DFM-CIN are the complexes identified by TSN-PCD, and three parameters *th*,*Smin*,_*Smax*_. The parameter *th* is used as a threshold of the similarity between two protein complexes, the parameters _*Smin*_and _*Smax*_in algorithm DFM-CIN are used to control the size of the identified functional modules, which are developed to make users get functional modules of suitable size, depending on their own requirements. The output of DFM-CIN is the final identified functional modules.

It is well known that functional modules are closely related to protein complexes. In previous studies, most clustering algorithms do not distinguish between them. However, their biological meanings are very different. In one the processes occur at the same time, while in the other, they occur at different times. According to the close relationship and different biological meaning between functional modules and protein complexes, we propose a new algorithm DFM-CIN for detecting functional modules based on the complex-complex interaction network. In the complex-complex interaction network, each vertex represents a protein complex and each edge represents the relationship of two protein complexes. The description of algorithm DFM-CIN is shown in Figure 4. To describe more simply, some related definitions are given as following.

For a protein complex _*Ci*_, let $T_{C_{i}}$ be the set of times that protein complex _*Ci*_functions in the corresponding TSNs. If two protein complexes function at least in one same time (ie., that, $T_{C_{i}}\cap T_{C_{j}}\neq \emptyset$), we say that these two protein complexes are synchronous. If two protein complexes function in two continuous times, (ie., that, $T_{C_{i}}\nabla T_{C_{j}}\neq \emptyset$), we say that these two protein complexes are adjacent to each other.

Let graph *G*(*V*,*E*) denote the complex-complex interaction network (abbreviated to CIN). In graph *G*, a vertex represents a protein complex, an edge represents a connection between two protein complexes, and the edge weight represents how similar two protein complexes are. There is a one-to-one correspondence between a protein complex _*Ci*_ and a vertex _*vi*_ of *G*.

For a weighted graph *G*, the weighted degree of a vertex *v* is donated as _*dw*_(*v*) , which is the sum of weights of the edges connecting *v*.

(2) $$d_{w}(v)=\underset{u\in N_{v};(u,v)\in E}{\sum }w(u,v).$$

For a vertex *v* in a subgraph K⊆G, its weighted in-degree, denoted as $d_{w}^{\mathrm{in}}(K,v)$, is the sum of the weights of edges connecting vertex *v* to other vertices belonging to *K*, and its weighted out-degree, denoted as $d_{w}^{\mathrm{out}}(K,v)$, is the sum of the weights of edges connecting vertex *v* to other vertices in the rest of the graph *G*. $d_{w}^{\mathrm{in}}(K,v)$ and $d_{w}^{\mathrm{out}}(K,v)$ can be formed as follows:

(3) $$d_{w}^{\mathrm{in}}(K,v)=\underset{u,v\in K;(u,v)\in E}{\sum }w(u,v).$$

(4) $$d_{w}^{\mathrm{out}}(K,v)=\underset{v\in K;u\notin K;(u,v)\in E}{\sum }w(u,v).$$

It is clear that the weighted degree _*dw*_(*v*) of a vertex *v* is equal to the sum of $d_{w}^{\mathrm{in}}(K,v)$ and $d_{w}^{\mathrm{out}}(K,v)$.

The modularity _*MK*_of a subgraph K⊆G is defined as follows:

(5) $$M_{K}=\frac{\Sigma_{v\in K}d_{w}^{\mathrm{in}}(K,v)}{\Sigma_{v\in K}d_{w}^{\mathrm{in}}(K,v)+\Sigma_{v\in K}d_{w}^{\mathrm{out}}(K,v)}$$

The inputs of algorithm DFM-CIN are protein complexes and their frequencies. First, each protein complex is condensed into a vertex. If two protein complexes _*Ci*_and _*Cj*_ are synchronous or adjacent, and their similarity is equal to or larger than *th*, an edge is added to vertex _*vi*_ and vertex _*vj*_. The similarity _*SC*_(_*Ci*_,_*Cj*_) of two complexes _*Ci*_and _*Cj*_ is defined as:

(6) $$S_{C}(C_{i},C_{j})=\frac{\left|C_{i}\cap C_{j}\right|}{\sqrt{\left|C_{i}|\times |C_{j}\right|}}$$

Based on the evaluation of similarity among protein complexes, a weighted graph *G*(*V*,*E*) is constructed with vertices representing protein complexes and edges representing connections among protein complexes. Then, all vertices of graph *G* are sorted into queue *Q* in non-increasing order in terms of their corresponding complexes’ frequencies. After that, algorithm DFM-CIN initializes a set *R* to store the identified modules. Next, the first vertex in queue *Q* is selected as a seed and then initialed as a singleton cluster *K*. Then, algorithm DFM-CIN extends cluster *K* by gradually adding its neighbors based on the evaluation of their contributions to _*MK*_. The neighbors of a cluster *K* are a collection of the neighbors of all vertices in *K*. For a neighbor vertex _*vi*_, its contribution to the modularity _*MK*_of cluster *K* is defined as:

(7) $$\delta_{K}^{M}(v_{i})=M_{K\cup \{v_{i}\}}-M_{K}$$

If any neighbors make positive contributions, the neighbor that has the maximum $\delta_{K}^{M}$ is added into *K*. Then, the neighbors of *K* are updated and another round of evaluation is performed. If no neighbors of *K* make positive contributions, set _*NK*_=*∅*. The extension performed on a cluster *K* will stop when _*NK*_=*∅*. A new functional module is generated simultaneously. At the same time, all vertices in the identified functional module are removed from queue *Q*. For each loop, algorithm DFM-CIN always selects the first vertex in queue *Q* as the seed and extends from it. The whole extending processes will stop when the queue *Q* is null. The parameters _*Smin*_ and _*Smax*_ in algorithm DFM-CIN are used to control the size of the identified functional modules, which are developed to make users get functional modules of suitable size, depending on their own requirements.

**Figure 5:**
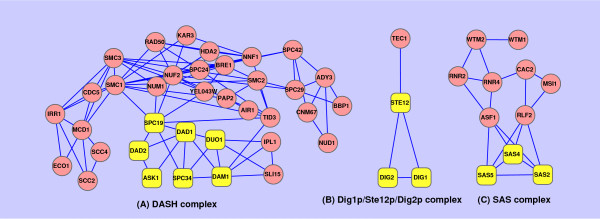
**Examples of protein complexes identified by TSN-PCD in a dynamic network and those identified by HC-PIN in a static network.** Figure 5 provides three examples of proteins complexes identified by TSN-PCD in a dynamic network and those corresponding complexes identified by HC-PIN in a static network. The circle and round rectangle vertices both represent the proteins of complexes identified by HC-PIN in a static network. The round rectangle vertices represent the proteins of complexes identified as TSN-PCD in dynamic network that are matched perfectly by known protein complexes. The known protein complexes are (**A**) DASH complex; (**B**) Dig1p/Ste12p/Dig2p complex; (**C**) SAS complex.

## Results and discussion

### Datasets and evaluation methods

The original protein-protein interaction data of *S.cerevisiae*, consisting of 4950 proteins and 21,788 interactions, was downloaded from the DIP database (2009, version12) [35]. The gene-expressing profiles of *S.cerevisiae* were retrieved from Tu et al., 2005[36], which contains 6777 gene products and 36 samples in total, with 4,858 genes involved in the yeast PPI network. We integrated gene expression profiles with the PPI network to construct a series of time-sequenced subnetworks (TSNs). In the integration process, the gene products with an expression value lower than 0.7 are filtered.

In an effort to evaluate the proposed algorithms of TSN-PCD and DFM-CIN, we compared them with five previously proposed clustering algorithms: MCL[32,33]), MCODE[3], CPM[34], COACH[9], and SPICI[10]. MCL is a fast and highly scalable clustering algorithm for networks based on stochastic flow, and its superiority for the extraction of protein complexes has been proven by Brohee *et al*[37]. MCODE is a typical density-based local search algorithm. CPM is an algorithm for detecting overlapping communities in biological networks [34], and formed the basis for a famous tool called CFinder [38]. COACH and SPICI are the two most recent algorithms for clustering PPI networks to discover protein complexes and functional modules. The values of the parameters in each algorithm are selected from those recommended by the authors.

### Identification of protein complexes in dynamic protein-protein interaction network

First of all, the proposed algorithm TSN-PCD is applied to the dynamic PPI network of *S.cerevisiae*. There are 865 different protein complexes detected, and ∼60*%* of the protein complexes are explored in only one TSN and ∼24*%*are discovered in more than three TSNs. So many protein complexes are only found in one TSN. This may be caused by the strict definition of protein complexes. For the complexes, they will be considered as two different complexes even they have most common proteins. How to deal with the overlapped protein complexes is an important and challenging issue. In future, we will study complexes over time-sequenced networks and investigate the relationship of the proteins in the protein complex. Moreover, the threshold value used to filter gene products at each time point may be another reason. Lower threshold of gene expression causes protein complexes tending to appear in less TSNs.

To directly validate the identified protein complexes, we compare them with the known protein complexes provided by the literature published in Nucleic Acids Research([39]). The 532 known protein complexes are regarded as the gold standard. Here, we use the same scoring scheme used in [3,8] to determine how effectively a predicted complex (*Pc*) matches a known complex (*Kc*). The overlapping score *OS*(*PcKc*) between a predicted complex *Pc*and a known complex *Kc*is calculated by the following formula:

(8) $$\mathrm{OS}(\mathrm{Pc},\mathrm{Kc})=\frac{|V_{\mathrm{Pc}}\cap V_{\mathrm{Kc}}{|}^{2}}{\left|V_{\mathrm{Pc}}|\times |V_{\mathrm{Kc}}\right|}$$

where |_*VPc*_| is the number of proteins in *Pc* and |_*VKc*_| is the number of proteins in *Kc*, and $\left|V_{\mathrm{Pc}}\cap V_{\mathrm{Kc}}\right|$ is the number of common proteins both in *Pc* and in *Kc*. A known complex and a predicted complex are considered as a match if their overlapping score is equal to or larger than a specific threshold. Our analysis based on different overlapping score thresholds (from 0 to 1 with a 0.1 increment) shows that the number of matched known complexes of TSN-PCD clustering in a dynamic network is consistently higher than that of HC-PIN clustering in a static network, which implies that the dynamic network is more suitable to exploring protein complexes, as it can reflect the dynamics of the network. In Figure 5, three examples are given to show how TSN-PCD identifies protein complexes more accurately than HC-PIN does in a static network.

**Figure 6:**
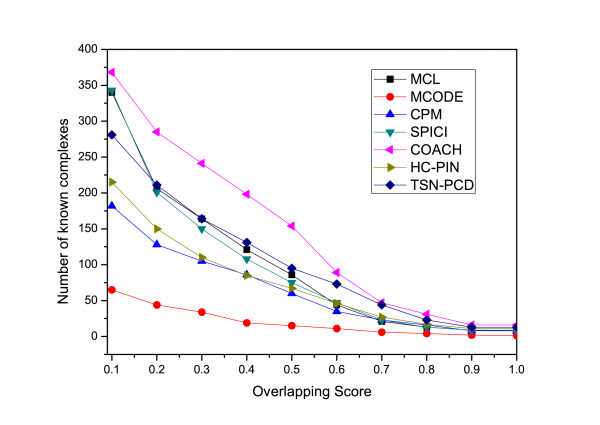
**Comparison of the percentage of matched complexes identified by TSN-PCD and that by COACH.** Though, the complexes predicted by COACH can match more known complexes than that of TSN-PCD as shown in Figure 7, TSN-PCD has larger percentage of matched predicted complexes than that of COACH with respect to each overlapping score.

**Figure 7:**
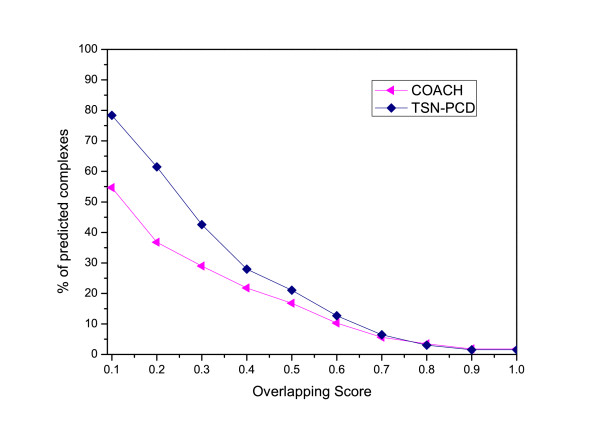
**Comparison of the number of known complexes matched by the predicted protein complexes by TSN-PCD and other five algorithms MCL, MCODE, CPM, COACH, SPICI.** Figure 7 shows the number of matched known complexes with respect to different overlapping scores for different sets generated by TSN-PCD and other five algorithms: MCL, MCODE, CPM, COACH, SPICI. TSN-PCD predicts the protein complexes from dynamic network while the others identify the protein complexes from the static network. The results show that TSN-PCD and COACH can match more known protein complexes than the other algorithms.

As shown in Figure 5, DASH complex, Dig1p/Ste12p/Dig2p complex, and SAS complex are perfectly located by TSN-PCD in a dynamic network. However, these three known protein complexes are enclosed in larger clusters detected by HC-PIN in a static network. These phenomena illustrate that topological information alone is not enough to discover different complexes, and that the time-sequenced gene expressions are useful for correct identification of protein complexes which function at different times. Table 1 also gives several examples of known protein complexes which are neither perfectly identified by HC-PIN nor ideally discovered by TSN-PCD. The pair of standard names and systematic names in Table 1 can be seen in Additional file 2. But the results of TSN-PCD are closer to the target than that of HC-PIN. Take the DASH complex for example: its 7 proteins are included in a 36-member cluster detected by HC-PIN. In contrast, TSN-PCD identifies a 7-member cluster which also includes the same proteins. Of course, there is a collection of protein complexes which are detected both by TSN-PCD and by HC-PIN correctly and for some special cases, a cluster of HC-PIN may match better than that of TSN-PCD. The matched results of all the protein complexes of HC-PIN and TSN-PCD can be seen in Additional file 3.

**Table 1:** Examples of protein complexes identified by TSN-PCD more precisely in a dynamic network than that identified by HC-PIN in a static network

		**Best matched complexes identified**			**Best matched complexes identified**		
**Known protein complexes**		**in static network (HC-PIN)**			**in dynamic network (TSN-PCD)**		
**Complexes**	**Proteins**	**#. Pc**	**Size(Overlap)**	**OS**	**#. Pc**	**Size(Overlap)**	**OS**
Dig1p/Ste12p/Dig2p complex	DIG1;DIG2;STE12	43	4(3)	0.75	302	3(3)	1.00

AP-3 adaptor complex	APL5;APL6;APM3;	109	3(3)	0.75	77	4(4)	1.00
	APS3						
SAS complex	SAS2;SAS4;SAS5	122	11(3)	0.27	404	3(3)	1.00
MRX complex	MRE11;RAD50;XRS2	39	4(1)	0.083	218	3(3)	1.00
prefoldin complex	GIM3;GIM4;GIM5;	8	11(5)	0.45	107	6(5)	0.83
	PAC10;PFD1;YKE2						
AP-1 adaptor complex	APL2;APL4;APM1;	116	6(4)	0.67	401	5(4)	0.8
	APS1						
DASH complex	ASK1;DAD1;DAD2;	56	36(7)	0.15	857	7(7)	0.78
	DAD3;DAD4;DAM1						
	DUO1;SPC19;SPC34						
FBP degradation complex	RMD5;GID7;GID8;	83	4(3)	0.28	586	8(7)	0.77
	VID24;VID28;VID30						
	FYV10;YDL176W						
ARGR complex	ARG80;ARG81;ARG82;	26	6(4)	0.67	158	3(3)	0.75
	MCM1						
retromer complex	PEP8;VPS29;VPS35	72	5(3)	0.6	45	4(3)	0.75
alpha DNA polymerase:primase complex	POL1;POL12;PRI1;	110	12(3)	0.19	709	3(3)	0.75
	PRI2						
Sec62p/Sec63p complex	SEC62;SEC63;SEC66;	75	129(1)	0.002	584	3(3)	0.75
	SEC72						
Kornberg’s mediator (SRB) complex	SSN3;SSN8;SRB8;	50	54(20)	0.30	552	19(18)	0.68
	SSN2;CSE2;GAL11						
	MED1;MED11;MED2						
	MED4;MED6;MED7						
	MED8;NUT1;NUT2						
	PGD1;RGR1;ROX3						
	SIN4;SRB2;SRB4						
	SRB5;SRB6;SRB7						
	SOH1						

Moreover, we compare other five algorithms MCL, MCODE, CPM, SPICI, COACH with TSN-PCD by matching their predicted protein complexes with the 532 known complexes. The comparison results show that TSN-PCD and COACH can identify more known protein complexes than MCL, MCODE, SPICI, CPM. As COACH gets such a good recall from known complexes, we compared its predicted complexes with that of TSN-PCD. As shown in Figure 6, TSN-PCD has a better score when considering the percentage of predicted complexes over each overlapping score.

**Figure 8:**
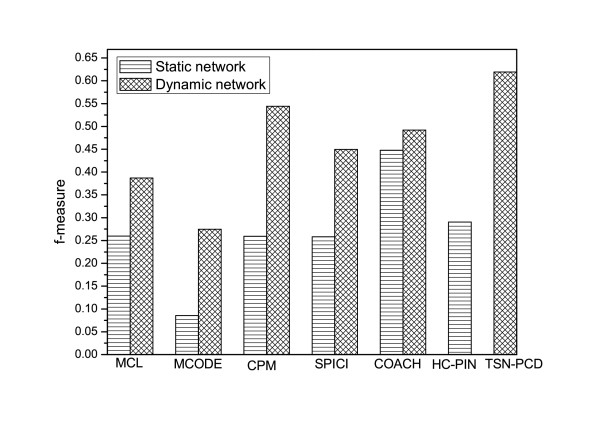
**Comparison of ***f ***-measure of TSN-PCD and that of other algorithms performed on static and dynamic protein-protein interaction networks.** To evaluate the effectiveness of TSN-PCD for identifying protein complexes, the *f *-*measure* results of HC-PIN performed on static network and those of five other protein complex discovery algorithms: MCL, MCODE, CPM, SPICI, and COACH performed on static and dynamic PPI networks are also shown in Figure 8.

To further estimate the performance of TSN-PCD for detecting protein complexes, a comprehensive evaluation method called *f *-*measure* is used. For a clustering algorithm, its *f *-*measure* is defined as a harmonic mean of its sensitivity (*Sn*) and specificity (*Sp*).

(9) $$\text{f}-\mathrm{measure}=\frac{2*\mathrm{Sn}*\mathrm{Sp}}{\mathrm{Sn}+\mathrm{Sp}}$$

(10) $$\mathrm{Sn}=\frac{\mathrm{TP}}{\mathrm{TP}+\mathrm{FN}}$$

(11) $$\mathrm{Sp}=\frac{\mathrm{TP}}{\mathrm{TP}+\mathrm{FP}}$$

where *TP* (true positive) is the number of the predicted complexes (*Pc*) matched by the known complexes (*Kc*), *FP* (false positive) equals the total number of *Pc* minus *TP*, and *FN* (false negative) is the number of *Kc* that are not matched by *Pc*.

The *f *-*measure* results of TSN-PCD and five other algorithms (MCL, MCODE, CPM, SPICI and COACH) performed on static and dynamic PPI networks are shown in Figure 8. From Figure 8 we can see that the *f *-measure of TSN-PCD is much higher than that of HC-PIN, MCL, MCODE, CPM, SPICI and COACH on a static PPI network. The *f *-measure of TSN-PCD is about two times more than that of MCL, CPM, and SPICI, and it is about six times more than that of MCODE performed on the static network. As TSN-PCD is applied in a dynamic network and MCL, MCODE, CPM, SPICI and COACH are applied in a static network, it is difficult to confirm what really contributes to the improvement of *f *-*measure* of TSN-PCD, TSN-PCD itself or the dynamic network? Therefore, we also apply another five algorithms (MCL, MCODE, CPM, SPICI and COACH) to the dynamic network. That is, we replace the subroutine HC-PIN of TSN-PCD with MCL, MCODE, CPM, SPICI and COACH, respectively. The comparison of the *f *-measure results of TSN-PCD with those of the other five algorithms when applied to a dynamic network are also shown in Figure 8. The *f *-*measure* values of MCL, MCODE, CPM, SPICI and COACH applied to a dynamic network are improved relative to those obtained when static network was used. From Figure 8 we can also find that the *f *-*measure* of TSN-PCD is consistently higher than that of MCL, MCODE, CPM, SPICI, and COACH, even when performed on a dynamic network. The results show that not only the use of a dynamic network, but also the algorithm, TSN-PCD, enhances the accuracy of identifying protein complexes. Algorithm TSN-PCD outperforms all five previous algorithms in the detection of protein complexes.

**Figure 9:**
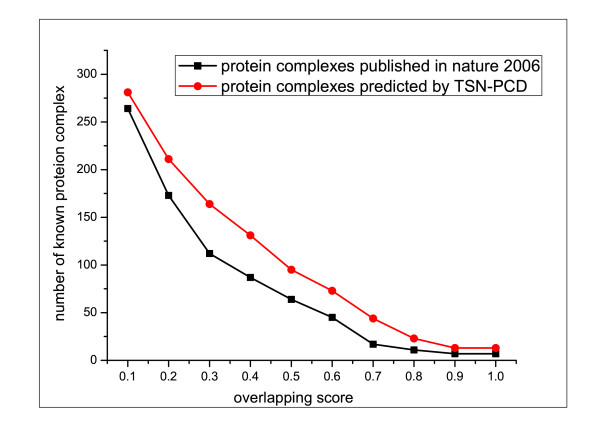
**Comparison of the protein complexes predicted by TSN-PCD and that published in Gavin’s publication(Nature 2006).** Figure 9 shows the number of matched known protein complexes with respect to different overlapping scores which rang from 0.1 to 1.0. The protein complexes predicted by TSN-PCD and that published in Gavin’s publication(Nature 2006) are matched with the known complexes, respectively. As shown in Figure 9, more known protein complexes are matched by the predicted complexes of TSN-PCD than that of Gavin’s.

In [40], Gavin et al. also provided 491 protein complexes which were determined by using affinity purification and mass spectrometry. By comparing the 865 complexes identified by our method TSN-PCD with Gavin’s 491 complexes, we surprisingly found that not any predicted complexes of TSN-PCD were the same as that of Gavin’s. Only 41 complexes of TSN-PCD were similar(*OS*≥0.5) to that of Gavin’s. As there is such a low overlapping between the TSN-PCD and the Gavin’s, we matched them with the known complexes, respectively. The comparison results are shown in Figure 9. From Figure 9 we can find that more knwon protein complexes are matched by the predicted complexes of TSN-PCD than that of Gavin’s.

### Evaluating functional modules based on Function Enrichment

It is well known that functional modules are closely related to protein complexes. In fact, most clustering algorithms detect both protein complexes and functional modules without distinguishing between the two. In this paper, we constructed a weighted graph (CCI network) by calculating the similarities among the identified protein complexes and analyzing their relationships in time. Then, the proposed algorithm DFM-CIN was applied to the weighted graph to discover functional modules. The similarity threshold *th*=0.5 is used here. The effect of its variation will be discussed later. In the following, 0.5 is used as a default value for the algorithm, DFM-CIN, if without special instructions.

To get insights on the shared, underlying biological processes of the identified functional modules, we use Gene Ontology annotations, downloaded from the Saccharomyces Genome Database (SGD) [41], to analyze their enrichments. Most of the identified functional modules appear to be enriched for proteins related to the same or similar biological processes. For example, all 5 proteins in module 175 function as “protein deneddylation”, all 9 proteins in module 62 belong to “cyclin catabolic process”, all 58 proteins in module 271 are related to “cellular macromolecule metabolic process”, out of which 57 proteins participate in “regulation of transcription, DNA-dependent”. To further test and verify the biological significance of the identified functional modules, we quantify their GO biological process term co-occurrences by using the SGD. For each identified functional module, its P-values are calculated. The smaller the P-value of a GO term, the more statistically significant the use of the GO term in the functional model[7,42,43]. The common cutoff of 0.001 is used here to differentiate between significant and insignificant groups. The lowest P-values of GO term of the 258 significant modules range from 7.85E-04 to 1.56E-66. The percentage of the significant functional modules, average −*log*(P-value), and the percentage of modules whose P-value falls within P<E-15, [E-15, E-10], [E-10, E-5], and [E-5, 0.001] are shown in Table 2.

**Table 2:** Functional enrichments of the identified complexes detected by TSN-PCD and functional modules detected by DFM-CIN, MCL, MCODE, CPM, SPICI, and COACH

	**Number of**	**Average**	**% of significant**	**Average**	**Significant modules(P)**			
**Algorithms**	**modules**	**size**	**modules**	**(-logP)**	**<E-15**	**E-15 to E-10**	**E-10 to E-5**	**E-5 to 0.001**
TSN-PCD	865	26.41	95.95	16.36	39.77%(344)	19.65%(170)	26.24%(227)	10.29%(89)
DFM-CIN	280	18.28	92.14	14.49	30.71%(86)	13.93%(39)	34.64%(97)	12.86%(36)
MCL	619	6.58	59.93	5.07	4.2%(26)	5.00%(31)	20.52%(127)	30.21%(187)
MCODE	50	15.66	92.00	8.53	12.00%(6)	12.00%(6)	50.00%(25)	18.00%(9)
CPM	126	11.59	92.65	11.14	20.63%(26)	14.28%(18)	38.89%(49)	19.84%(25)
SPICI	552	11.59	66.85	5.00	4.71%(26)	3.80%(21)	21.19%(117)	37.14%(205)
COACH	894	8.99	85.57	7.76	10.85%(97)	11.97%(107)	35.79%(320)	26.96%(241)

**Figure 10:**
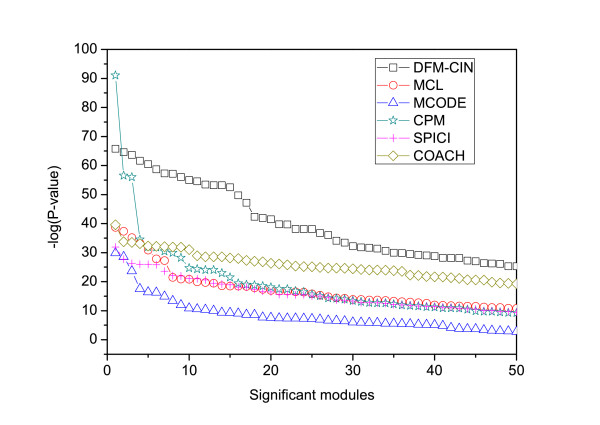
**Comparison of the P-value distribution of significant modules generated by DFM-CIN and those detected by other algorithms.** The *x* axis represents the number of significant modules, and the *y* axis represents the -log(P-value) for each corresponding module. The 50 most significant modules identified by our algorithm DFM-CIN are consistently more significant than those generated by other algorithms.

As shown in Table 2, the percentage of significant modules detected by DFM-CIN is similar to that identified by MCODE and CPM, but much greater than that generated by MCL, SPICI, and COACH. Moreover, the average -*log*(P-value) of DFM-CIN is much greater than that of the other five algorithms. The absolute percentage of functional modules identified by our algorithm, DFM-CIN, with P-values less than E-15 is 7 times more than that of SPICI and MCL, 2.5 times more than that of COACH and MCODE, and about 1.5 times than that of CPM. Figure 10 illustrates the P-value distributions of the significant modules generated by all these algorithms. As shown in Figure 10, the 50 most significant modules identified by our algorithm DFM-CIN are consistently more significant than those generated by other algorithms. The statistical results from Table 2 and Figure 10 show that DFM-CIN is more effective for identifying functional modules than other algorithms.

### Effect of parameter *th* on the identification of functional modules

In this section, we will discuss the effect of parameter *th* on the identification of functional modules. The values of parameter *th* are set to be from 0.2 to 0.6 with 0.1 increments. In total, five different sets are obtained by variation of *th*. For each set, the number of identified functional modules, the average size, the percentage of significant modules, the average -*log*(P-value), and the percentage of modules whose P-value falls within P<E-15, [E-15, E-10], [E-10, E-5], and [E-5, 0.001] are shown in Table 3.

**Table 3:** Effect of parameter *th* on the identification of functional modules

**Parameter*****th***	**Number of modules**	**Average size**	**% of significant modules**	**Average (-logP)**	**Significant modules(P)**			
					**<E-15**	**E-15 to E-10**	**E-10 to E-5**	**E-5 to 0.001**
0.2	223	21.21	90.58	14.88	34.98%(78)	12.11%(27)	30.94%(69)	12.55%(28)
0.3	242	19.29	91.32	14.48	33.88%(82)	13.64%(33)	31.40%(76)	12.40%(30)
0.4	261	17.78	91.57	14.01	30.65%(80)	16.92%(42)	32.57%(85)	12.26%(32)
0.5	280	18.28	92.14	14.49	30.71%(86)	13.93%(39)	34.64%(97)	12.86%(36)
0.6	320	19.22	93.12	14.82	31.25%(100)	13.75%(44)	35.00%(112)	13.12%(42)

The number of the identified functional modules increases with the increase of *th*. This is because the larger value of *th* leads to fewer edges connecting the protein complexes. That is to say, a sparser graph is constructed by using a larger value of *th*. As a result, more functional modules will be identified with the same criterion for generating modules. From Table 3, we can see that DFM-CIN is not very sensitive to the input parameter, *th*, for evaluation of its biological meaning.

### Relationship between protein complexes and functional modules

**Figure 11:**
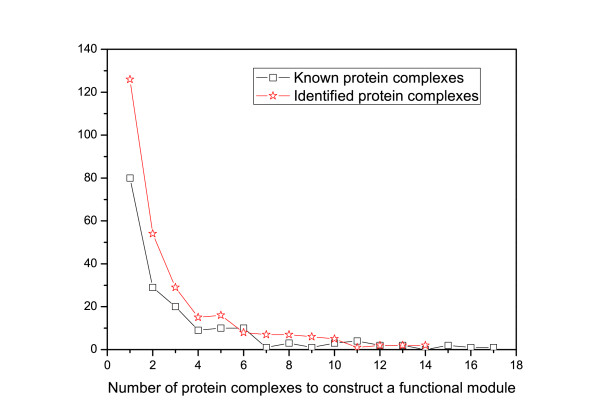
**The relationship between protein complexes and functional modules.** The *x* axis represents the number of protein complexes to construct a functional module while the *y* axis represents the number of modules. To avoid the bias of using the algorithm DFM-CIN, the relationship between the known protein complexes and the identified functional modules is also shown in Figure 9.

As protein complexes and functional modules are significantly related to each other, we discuss their relationships in this section. Analysis of the identified functional modules shows that they generally include multiple protein complexes. As shown in Figure 11, ∼55*%*of the functional modules consist of at least two identified protein complexes. To avoid the bias of using the algorithm DFM-CIN, we also analyze how many known protein complexes a functional module will include. Given a known protein complex (Kc) and an identified functional module (Im), we say that Kc is part of Im if more than 60% proteins in Kc are members of Im. The results agree closely with the identified protein complexes, as shown in Figure 11. Another key feature of the relationship between protein complexes and functional modules is that the complexes included in the same module generally participate in the same biological process.

There are about 45% identified functional modules which consist of only one protein complex. For example, module (*#*15) and module (*#*235) both consists of only one protein complex (The identified functional modules are available from Additional file 4). Module *#*15 functions as “nuclear-transcribed mRNA catabolic process, exonucleolytic, $3^{\prime \prime }-5^{\prime \prime }$” which includes a Nonsense-mediated mRNA decay pathway complex. In the definition of GO:0000184, the nonsense-mediated decay pathway for nuclear-transcribed mRNAs degrades mRNAs in which an amino-acid codon has changed to a nonsense codon. This prevents the translation of such mRNAs into potentially harmful, truncated proteins. Module *#*235, including a FBP degradation complex and a protein “MOH1”, whose function is unknown, participates in the process of “negative regulation of gluconeogenesis”.

**Figure 12:**
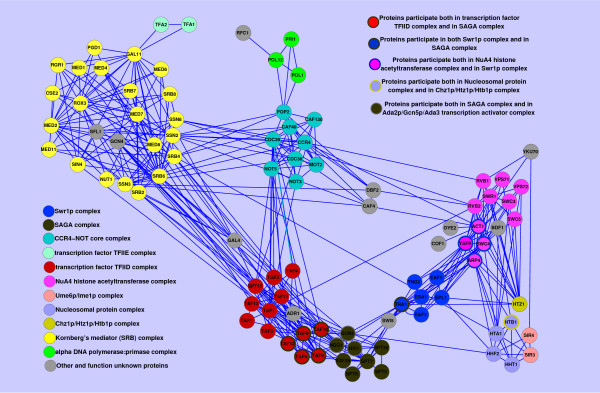
**An example of functional module identified from a complex-complex interaction network.** There are 11 known protein complexes contained in it. The 11 protein complexes are: Sw1p complex, SAGA complex, CCR4-NOT core complex, transcription factor TFIIE complex, transcription factor TFIID complex, NuA4 histone acetyltransferase complex, Ume6p/Ime1p complex, Nucleosomal protein complex, Chz1p/Htz1p/Htb1p complex, Kornberg’s mediator (SRB) complex, alpha DNA polymerase:primase complex.

For a functional module consisting of multiple protein complexes, exploration of its biological processes shows that these multiple protein complexes participate in the same biological process. The illustrated example module *#*166, and the protein complexes contained in it are shown in Figure 12. The biological process of module *#*166 is “regulation of transcription” with the lowest P-value=2.55E-68. There are 13 protein complexes, in total, with different sizes recovered by module *#*166. The biggest one is Kornberg’s mediator (SRB) complex, which has been found to support activated transcription in yeast[44]. The transcription factor TFIID complex and SAGA complex are both multi-subunit complexes involved in transcription by RNA polymerase II [44,45]. As shown in Figure 12, there is an overlap between SAGA and TFIID. The common subset of SAGA and TFIID have been verified to be TBP-associated factors (TAFs) subunits which mediate a common function in global transcription [46]. NuA4 histone acetyltransferase complex is active in transcription and DNA repair[47]. Four proteins of NuA4 histone acetyltransferase complex are also found in Swr1p complex. The general transcription factor TFIIE complex, though only composed of two proteins, plays important roles at two distinct, but sequential steps, in transcription as follows: preinitiation complex formation and activation (open complex formation), and the transition from initiation to elongation[48]. The CCR4-NOT complex functions as general transcription regulation complex[49]. The alpha DNA polymerase:primase complex catalyzes the synthesis of an RNA primer on the lagging strand of replicating DNA (annotated in GO:0005658). Moreover, the conversion of Ume6p from a repressor into an activator by association with the meiotic inducer Ime1p is required in meiotic induction[50]. According to the above analysis, the different functional components of module *#*166 participate in the mechanism of transcription regulation. More functional modules which consist of multiple protein complexes can be seen in Additional file 5.

## Conclusion

An important and challenging task in post-genomic era is to investigate the systematic and dynamic organization of PPI networks and explore biologically significant clusters. This paper introduces a new framework for constructing a dynamic PPI network by integrating gene expression data into PPI data. An important contribution of the framework is that in which protein complexes and functional modules can be distinguished. Few such works have been done before, though many researchers know that protein complexes and functional modules are two different concepts which have different biological meanings. In the proposed framework, the dynamic PPI network is composed of a series of time-sequenced subnetworks, based on the the time that the interactions are activated. Two different clustering algorithms: TSN-PCD and DFM-CIN are proposed for identifying protein complexes and functional modules, respectively.

To test and validate the effectiveness of the proposed framework and clustering algorithms, the identified protein complexes and functional modules are compared with those detected by other clustering algorithms: MCL, MCODE, CPM, SPICI, and COACH. A quantitative comparison based on *f*-*measure* reveals that our algorithm TSN-PCD outperforms the other five protein complex discovery algorithms. Comparison of the results on static and dynamic PPI networks shows that the combination of temporal gene expression data with PPI data is worthwhile for protein complex discovery.

An evaluation of the identified functional modules involved the function enrichment. The evaluation shows that the identified functional modules discovered by DFM-CIN are statistically significant in terms of “Biological Process”. More importantly, the analysis of the relationship between protein complexes and functional modules reveals that a module generally consists of one or more protein complexes, and the protein complexes contained in the same module participate in the same biological process universally.

In conclusion, the proposed framework and clustering algorithms, TSN-PCD and DFM-CIN, effectively reveals modular organization of the PPI network, and they distinguish well between protein complexes and functional modules.

## Competing interests

The authors declare that they have no competing interests.

## Author’s contributions

ML and XW developed and implemented the new framework and algorithms. ML drafted the manuscript under the guidance and supervision of JW and YP. All authors read and approved the final manuscript.

## Supplementary Material

Addtional file 1 **Protein complexes identified by TSN-PCD and their frequencies.** Additional file 1 provides the protein complexes identified by the algorithm TSN-PCD from the dynamic protein interaction network. The frequency of each identified protein complex and the information of subnetworks in which the protein complex is included are also shown in the Additional file 1. Click here for file

Addtional file 2 **A supplemental table with the standard names and systematic names paired.** Additional file 2 provides a supplement table with standard names and systematic names paired for Table 1, Figure 5 and Figure 12. Click here for file

Addtional file 3 **The matched results of the identified protein complexes of HC-PIN and TSN-PCD with known complexes.** Additional file 3 provides the results of the complexes predicted by HC-PIN and TSN-PCD matched with the known complexes, respectively. Click here for file

Addtional file 4 **Functional modules identified by DFM-CIN.** Additional file 4 provides the functional modules identified by the algorithm DFM-CIN. Click here for file

Addtional file 5 **Identified functional modules consist of multiple complexes and their relationship.** Additional file 5 provides all the identified functional modules. For each module, we provide its possible function by p-value and its proteins, frequency, p-value and detail complexes it contains. Click here for file
